# Exploration of Potent Antiviral Phytomedicines from Lauraceae Family Plants against SARS-CoV-2 Main Protease

**DOI:** 10.3390/v14122783

**Published:** 2022-12-14

**Authors:** Himashree Bora, Madhu Kamle, Hesham Hassan, Ahmed Al-Emam, Sidharth Chopra, Nikhil Kirtipal, Shiv Bharadwaj, Pradeep Kumar

**Affiliations:** 1 Applied Microbiology Laboratory, North Easter Regional Institute of Science and Technology, Nirjuli 791109, Arunachal Pradesh, India; 2 Department of Pathology, College of Medicine, King Khalid University, Abha 62529, Saudi Arabia; 3 Department of Pathology, Faculty of Medicine, Assiut University, Assiut 71515, Egypt; 4 Department of Forensic Medicine and Clinical Toxicology, Faculty of Medicine, Mansoura University, Mansoura 35516, Egypt; 5 Division of Molecular Microbiology and Immunology, CSIR-Central Drug Research Institute, Sector 10, Janakipuram Extension, Sitapur Road, Lucknow 226031, Uttar Pradesh, India; 6 School of Life Sciences, Gwangju Institute of Science and Technology (GIST), Gwangju 61005, Republic of Korea; 7 Department of Biotechnology, Institute of Biotechnology, College of Life and Applied Sciences, Yeungnam University, 280 Daehak-Ro, Gyeongsan 38541, Republic of Korea; 8 Laboratory of Ligand Engineering, Institute of Biotechnology of the Czech Academy of Sciences v.v.i., BIOCEV Research Center, 25250 Vestec, Czech Republic; 9 Department of Botany, University of Lucknow, Lucknow 226007, Uttar Pradesh, India

**Keywords:** SARS-CoV-2, main protease, cassameridine, laetanine, litseferine, cassythicine, docking, MD simulations

## Abstract

A new Coronaviridae strain, Severe Acute Respiratory Syndrome Coronavirus-2 (SARS-CoV-2), emerged from Wuhan city of China and caused one of the substantial global health calamities in December 2019. Even though several vaccines and drugs have been developed worldwide since COVID-19, a cost-effective drug with the least side effects is still unavailable. Currently, plant-derived compounds are mostly preferred to develop antiviral therapeutics due to its less toxicity, easy access, and cost-effective characteristics. Therefore, in this study, 124 phytochemical compounds from plants of Lauraceae family with medicinal properties were virtually screened against SARS-CoV-2 M^pro^. Identification of four phytomolecules, i.e., cassameridine, laetanine, litseferine and cassythicine, with docking scores −9.3, −8.8, −8.6, and −8.6 kcal/mol, respectively, were undertaken by virtual screening, and molecular docking. Furthermore, the molecular dynamic simulation and essential dynamics analysis have contributed in understanding the stability and inhibitory effect of these selected compounds. These phytomolecules can be considered for further in vitro and in vivo experimental study to develop anti-SARS-CoV-2 therapeutics targeting the main protease (M^pro^).

## 1. Introduction

COVID-19 is a pandemic that has academics and scientists determined to developing new therapeutic tactics and plans to combat this catastrophic pandemic as soon as possible [1]. Currently, there are no specific therapeutic options for the virus, and treatment is based on symptoms and the repurposing of antiviral medicine [2].

In one study, the virtual screening of a library of FDA-approved medications revealed three promising macrocyclic antibiotics, polymyxin B, bafilomycin A, and rifampicin, that show promising and consistent in silico binding to more than one protein target of SARS-CoV-2. In contrast, other tested antimicrobials that belong to different categories, such as antituberculosis drugs or antiprotozoal drugs, did not show comparable affinity against the same targets [3].

There is another approach to find the plant based drug due to their low side effects, traditional medicinal plants’ less toxic natural compounds with antioxidant and antibacterial capabilities have been used often for thousands of years to cure a variety of illnesses [4].

So, There is a demand to find an alternative therapeutic methods for the development of novel vaccinations or some natural or plant based drug by using computational approach against COVID-19 [1].

Lauraceae, an important family of the plant kingdom rich in medicinal and aromatic plant species, possessed one of the most exceptionally aromatic genera, *Litsea sp*. This genus comprises 622 species dispersed across East Asia, New Zealand, the Tropics of Australia, and North and South America [5,6]. Plants belonging to the Lauraceae family are widely known for their medicinal value in maintaining human health [7]. Among others, *Litsea cubeba* is one of the oldest herbs, spicebush of economic importance, which is widely dispersed in East Asian countries like South China, Japan, and South Asian realms [8]. *Litsea* is an evergreen, fast-growing deciduous tree with a height of about 8 m. In India, it is found in the eastern belts of the Himalayas up to an altitude of 2700 m from sea level growing spontaneously in the Northeastern state of Assam, Manipur and Arunachal Pradesh. This plant is habitually called as “May Chang” or “Chinese pepper” in China, while in Assam, it is called “Mejankari” and in Arunachal Pradesh as “Taer” [9]. The entire plant of *Litsea cubeba* is highly aromatic, pungent and citrusy, which is one of the key features of this plant; thus, the berries are recommended in apothecary, aroma therapy and food [10]. The dried fruit of *L. cubeba* are used in Chinese medicine as Chen-Qie-Zi and other folk medicine as it is carmative, antiseptic, diuretic, sedative, used in the treatment of stomach hiccups, gastric cavity acroodynia, hernia neuralgia and congestion due to cold and cough [11]. The fruit is also known as the mountain spice used in many cuisines in Asian countries and also the EO of *Litsea cubeba* is broadly used in aromatherapy. Vast production of EO of *L. cubeba* occurs in China and almost 80% of the plant grows wildly across the country. According to Chen and Wang’s examination of annual export data, Europe accounts for the import of L. cubeba EO from China for more than 60% of the overall export at a volume-rate of 400 t/a at $4 million/a [12].

The phytochemical examination of Litsea sp. identified over 262 phytoconstituents, including fatty acids, lignans, butanolides and butenolactones, monoterpenes, triterpenes, and flavonoids [13]. The chemistry of *L.cubeba* EO principally comprises of more than 90% oxygenated monoterpenes, 30% of sesquiterpenes and 10% of monoterpene hydrocarbons. Citral, which is a combination of citral A (46%) and citral B (40%), dominates the fruit oil to a greater extent than other constituents like limonene (26%), linalool, -pinene, heptanone, and cintronellal do, while citronellol and linalool predominate in the bark and leaf oil [14]. The essential oil of *Litsea cubeba* is the secondary metabolite responsible for various biological activities including antibacterial, antifungal, insecticidal, antioxidant, anti-inflammatory, and anticancer properties [15].

The increasing need for new and improved antiviral drug lead us to evaluate several plant species having potential as novel antiviral agents because of the presence of a wide variety of active compounds [16]. For centuries folk medicines directed from plants have been used to treat people who suffer from viral infection. In the current situation when there is a new virus outbreak where no allopathic medicines work, people rely on herbal medicines as the only alternative. It is evident from the literature that essential oils are active against many DNA and RNA virues such as Polio virus, Herpes simplex virus (HSV-1 and HSV2), Dengue virus (DENV-1, DENV-2, DENV-3 and DENV-4), Influenza virus, Adenovirus, Junin virus, coxsackievirus B1 [17]. Since the disease’s outbreak and the WHO’s proclamation that it was a pandemic on 11 March 2020, there has been an increase in COVID-19 cases, raising analytical concerns on a global scale. The conquering of coronavirus 2 (SARS-CoV-2) into the host cells takes place by the virus spike glycoprotein (S protein) and human angiotensin-converting enzyme2 (hACE2) receptor, which is expressed in human organs [18]. The race for the cure is still on, and the phytoconstituent of many Indian Ayurvedic herbs turned out to be a vital therapeutic alternative for COVID-19 by targeting it’s S-protein [19,20]. Using molecular docking, Chikale and Sinha looked at two proteins, spike receptor-binding protein from SARS-CoV-2 and NSP15 endoribonuclease, to study the phytochemicals from Asparagus racemosus. Isolated molecules such as asparagine-C, asparagine-D, and asparagine-F were vulnerable to both proteins [21]. In disease propagation, the chief protease enzyme of SARS-CoV-2 (M^pro^) figures prominently by machining polypeptide, which is indispensable for viral replication and transcription. In *Withania somnifera* (Ashwagandha) four important molecules *viz.* Withanoside II, Withanoside IV, Withanoside V, Sitoindoside IX, and Somniferine, where Withanoside V and Somniferine turned out to have a strong binding affinity towards the protein active site with strong hydrogen bonds that inhibit the M^pro^ of SARS-CoV-2, indicating Aswagandha is a powerful antiviral agent [22,23]. In *Tinospora cordifolia* (Giloy) a compound named Berberine which is one of the main constituent of Giloy, showed as the best docked molecule which can also regulate the protease enzyme M^pro^ or 3CL^pro^ acting as an inhibitor with better stability towards CoV2 protein [18], similarly in tulsi (*Ocimum sanctum*) three compound namely Vicenin, Isorientin 4′-O-glucoside-2″-O-p-hydroxybenzoate and Ursolic acid act against M^pro^ of SARS-CoV-2 [24].

Likewise, *Litsea* being highly medicinal, showing potent biological activities against human diseases, the *Litsea* essential oil possessing incredible structural diversity is considered an excellent source of exploring diverse antiviral agents. *Litsea verticillate* was the first anti-HIV plant due to the presence of three compounds litseaverticillols L/M and Litseasesquibutenolide [25]. *Litsea japonica* is also effective against the Hepatitis E virus [26].

In addition to the well-known hepatoprotective silybin and other flavonoids and phenolic compounds, bioactive substances also include the antihypertensive alkaloid reserpine, potential anticancer drugs like paclitaxel, vincristine and vinblastine alkaloids, and a more significant cluster of glucosinolate glycosides that naturally occur in many pungent plants like mustard, cabbage, broccoli, rocket, and horseradish [27].

It has been reported that honey bee products containing potentially active chemical mixes have special features that may assist to protect, combat, and reduce symptoms of COVID-19 infection [28].

Shaldam et. al., suggested that the most effective substances on COVID-19 active sites included P-coumaric acid, ellagic acid, kaempferol, and quercetin (RdRb and M^pro^). These bioactive substances were also discovered to have potential antiviral activity against the human rhinovirus, which causes the common cold and is an RNA virus similar to SARS-CoV-2 [29].

So far, viral diseases have become a cardinal consternation for human well-being worldwide, and till now, only a few numbers of medications are available and effective against the number of viral strains.

*Lauraceae,* a family of medicinal plants with antiviral potential, has encouraged researchers to find a novel antiviral lead molecule. Keeping this view, the objective of our study is to explore the possibility of phytomolecules from Lauraceae family of plants to combat the Novel CORONA virus and provide a new source of cure to humankind.

## 2. Methodology

### 2.1. Receptor and Ligand Collection

The PDB database was used to get the SARS-CoV-2 M^pro^ structure’s three-dimensional (3D) crystal structure, which had a resolution of 2.16 (https://www.rcsb.org/ (accessed on 18 October 2020)) (PDB ID: 6LU7) [30]. Furthermore 124 phytochemical compounds from various plants of Lauraceae family were selected from PubChem Database (https://pubchem.ncbi.nlm.nih.gov/ (accessed on 18 October 2020)) [31,32] as ligand molecules for the screening analysis against the selected target protein.

### 2.2. Structure-Based Virtual Screening and Re-Docking Simulation

The MTiOpen Screen web server was utilised for structure-based virtual screening against SARS-CoV-2 M^pro^ to uncover potential inhibitors from the selected phytochemical compounds [33]. The receptor molecule was prepared before virtual screening by adding hydrogen atoms and by removing co-crystallized native ligand, heteroatoms, and solvent molecules using the Dock prep tool in USCF Chimera under the default parameters [34]. The native ligand binding residues (His^41^, Phe^140^, Cys^145^, Glu^166^, and Gln^189^) were provided to MtiOpen Screen server for calculation of grid for virtual screening. The highest four compounds were chosen for redocking, and intermolecular analysis with SARS-CoV-2 M^pro^ compared to reference ligand GC376 based on the high intensity of binding energy values obtained after the screening [35].

Re-docking studies were performed to determine how the inhibitors were bound to their target protein. The GC376, a dipeptide protease, was taken as the reference ligand in this study, showing inhibition against SARS-CoV-2 M^pro^ [35]. The binding pocket residues of SARS-CoV-2 M^pro^ with the reference ligand GC376 were selected to check the binding behaviour of compounds chosen with the target protein [35]. In Dock prep Chimaera, the polar hydrogen atoms and charges were added after the selected proteins, and other ligands were synthesised under the default option. By moving and changing the grid size around center co-ordinates (−13.539 × 18.826 × 63.171) at the binding site of SARS-CoV-2 M^pro^ [35], re-docking tests were carried out using AutoDock Vina under the default parameter [36]. Following redocking, the most advantageous ligand orientation for each molecule was picked for additional examination. Using Chimera’s energy reduction programme under default settings, all docked complexes had reduced energy consumption. Additionally, molecular 2D and 3D interaction images were produced using the ligand-receptor interaction module of the free academic Maestro (Schrödinger Release 2020-2: Maestro, Schrödinger, and Maestro).

### 2.3. Molecular Dynamics Simulation

Using the free Maestro-Desmond Interoperability, the selected target-ligand docked complexes were utilised to a 100 ns molecular dynamics (MD) simulation to examine stability and intermolecular interactions [37,38]. Using the protein preparation wizard of the Desmond-maestro interface, all protein-ligand contacts were preprocessed and improved. The system was configured for each complex using the TIP4P solvent model, orthorhombic box shape, and buffer box size calculation method. Salt and Na+ were also added to the mixture to inimized it. They were also eliminated from placements within 20. The Desmond minimization software was used to reduce the system model after the system was set up, with a maximum iteration limit of 5000 and a convergence criterion of 1.0 kcal/mol. The 100 ns MD simulation experiment was permitted to be run on the inimized system at the default settings. The OPLS-2005 force-field was used for md simulation of all the complexes.

### 2.4. Essential Dynamics and Dynamic Cross-Correlation Matrix (DCCM) Profiling

Analysis of correlated fluctuations for protein was done by application of Essential dynamics to disclose the motions that are of utmost requirement in the protein function. To collect the PCA (principal component analysis) on the respective MS simulation trajectory using Bio3d package, essential dynamics analysis was necessary to perform [29]. Furthermore, the correlation coefficient was also computed to review at which degree during MD simulation, residual displacements in docked protein were correlated by dynamic cross-correlation analysis in the Bio3d package [39]. To reduce the RMS (root mean square) differences between the equivalent residues of the structure, essential dynamics and dynamic cross-correlation matrix analysis was applied to all of the C-alpha atoms in the 5000 frames extracted from the 100 ns MD simulation trajectory and then superimposed to the initial pose. All the estimations for each trajectory of the respective complex were performed in the R program environment [40] with the Bio3d package.

## 3. Results and Discussion

### 3.1. Structure-Based Virtual Screening

**Figure 1:**
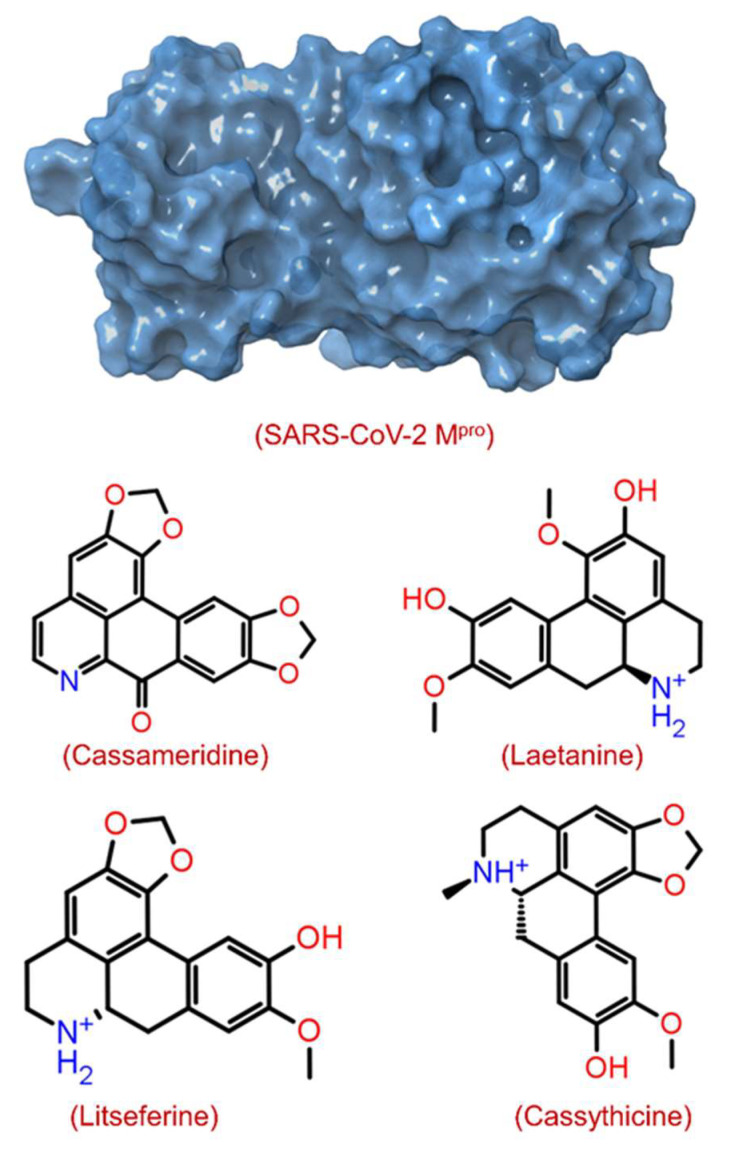
Structure of SARS-CoV-2 M^pro^ with 3D surface exhibiting various pockets and 2D chemical diagram for screened potential compounds as inhibitor of SARS-CoV-2 M^pro^.

The structural-based virtual screening (SBVS) technique searches the small molecules from a library to identify compounds most likely to bind to a receptor protein [41]. After the virtual screening experiment, the binding poses were evaluated to find out the best-docked complexes by re-docking protein and selected ligand molecules. In this communication, we used an SBVS technique to predict the binding potential of 124 compounds against SARS-CoV-2 M^pro^ with significant binding energy between −9.3 and −3.9 kcal/mol. The top 4 screened phytomolecules viz. cassameridine, laetanine, litseferine, and cassythicine (Figure 1 and Figure S1), were selected based on their binding energy. The docked complexes with the best binding poses of selected phytomolecules within the active pocket of SARS-CoV-2 M^pro^, were selected for protein-ligand complex preparation and intermolecular interaction analysis.

The binding energy observed for cassameridine, laetanine, litseferine and cassythicine, were −9.3, −8.8, −8.6, and −8.6 kcal/mol (Table 1 and Table S1)**,** respectively. However, in a recent study, doxycycline and minocycline antibiotics have been shown as potential inhibitor against SARS-CoV-2 M^pro^ with binding energy of >−7 kcal/mol [42]. In an in silico, the binding energy observed for Withanoside V, a natural compound from *Withania somnifera*, against SARS-CoV-2 M^pro^ was −8.96 Kcal/mol [22]. The binding energies observed in the above studies are higher than in the present study.

### 3.2. Re-Docking and Intermolecular Interaction Analysis

**Figure 2:**
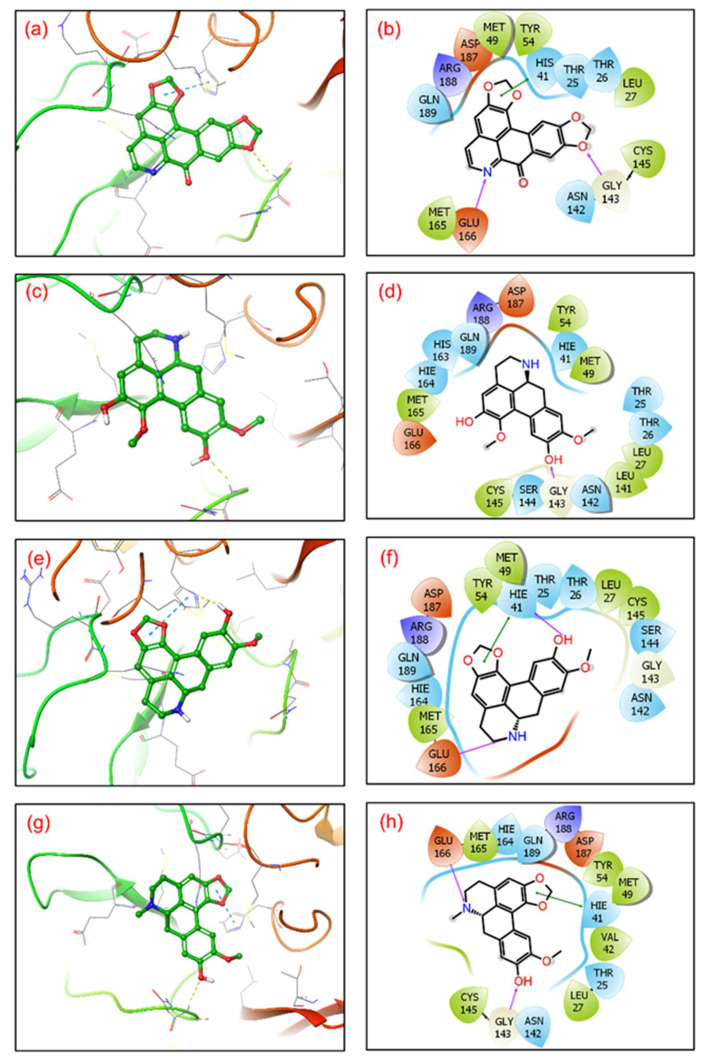
3D representation of SARS-CoV-2 docked complexes exhibiting intermolecular interactions with potential compounds; (**a**,**b**) Cassameridine, (**c**,**d**) Laetanine, (**e**,**f**) Litseferine, and (**g**,**h**) Cassythicine. Docked complexes with the active residues of the viral protease have been shown in a 2D interaction diagram as hydrogen bond formation (pink arrows), π-π interactions (green lines), hydrophobic (green), polar (blue), red (negative), violet (positive), and glycine (grey) interactions.

After molecular docking, molecular interaction analysis is essential to understand the forces and interactions providing strength and stability to the docked complexes [43,44]. Molecular interactions analysis of each protein-ligand complex showed various non-covalent interactions between SARS-CoV-2 M^pro^ and selected drug molecules, viz. cassameridine, laetanine, litseferine, and cassythicine. The reference ligand GC376 residual interaction with SARS-CoV-2 M^pro^ binding pocket were also studied at a 4 Å radius, along with the selected ligands. (Figure 2, Table 2).

The SARS-CoV-2 M^pro^-cassameridine complex exhibited interaction by two hydrogen bonds in the active region with Gly^143^ and Glu^166^ residues, respectively. The complex also revealed the π-π stacking interaction at residue His^41^. The interaction profiles of SARS-CoV-2 M^pro^ -laetanine reflected a single hydrogen bond formed with residue Glu^166^. Additionally, docked litseferine complex with SARS-CoV-2 M^pro^ displayed moderate hydrogen bonding with His41 and Glu166 residues, along with π-π stacking interaction at res0idue His^41^. In SARS-CoV-2 M^pro^-cassythicine docked complex two single hydrogen bonds at residues Gly^143^ and Glu^166^, were formed while His^41^ exhibited π-π stacking (Figure 2).

Additionally, hydrophobic, polar, positive and negative charge interactions with binding site residues were recorded in the SARS-CoV-2 M^pro^-phytochemical compound complex (Figure 2, Table 2). The re-docking and intermolecular interaction analysis of the selected compounds within the active pocket of viral protease suggested good molecular contacts with active residues and substrate binding residues. Notably, the confirmation of the binding pocket and interacting residues of SARS-CoV-2 M^pro^ are similar for the selected phytochemical compound and the reference compound GC376. Hence, computed docking scores and molecular contacts indicate the potential role of screened compounds in inhibiting viral protease, as reported for the GC376 inhibitor (Figure S2).

### 3.3. Molecular Dynamics Simulation Analysis

Molecular dynamics simulation (MDS) is a computative approach used to discover new drug lines to monitor the stability of molecular docked complexes over time [43,44]. In this study, root mean square deviation (RMSD) and root square mean fluctuation (RMSF) retrieved from corresponding 100 ns simulation trajectories were used to assess the stability of selected complexes. Usually, the structural variations necessary to determine the system’s dynamic stability are observed using RMSD and RMSF. To examine the stability of docked ligands at the active pocket of viral protease, intermolecular interactions between the protein and ligands were also estimated from the respective 100 ns simulation trajectories.

#### 3.3.1. RMSD and RMSF Analysis

**Figure 3:**
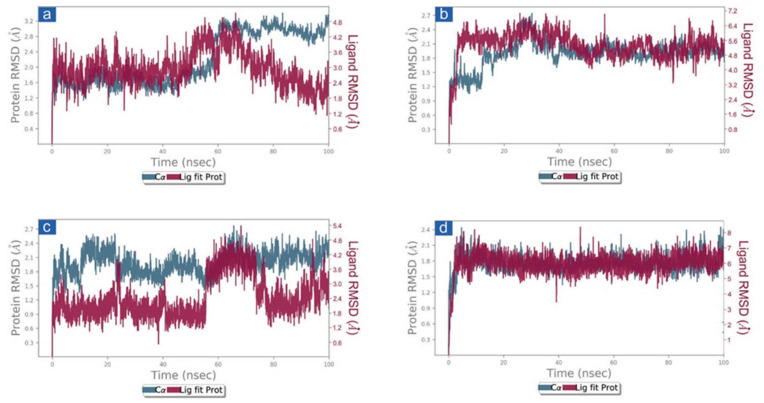
RMSD plots for SARS-CoV-2 M^pro^ docked with potential compounds, i.e., (**a**) Cassameridine, (**b**) Laetanine, (**c**) Litseferine, and (**d**) Cassythicine, extracted from 100 ns MD simulation.

First, the protein and ligand RMSD concerning the reference frame were examined in docked complexes of candidate drugs with SARS-CoV-2. With the exception of SARS-CoV-2 M^pro^-Cassythicine, the RMSD for SARS-CoV-2 M^pro^ showed deviations of <2.5 Å until 60 ns. This was followed by the state of equilibrium until the simulation’s conclusion (<3 Å). (Figure 3). These findings were also confirmed by the calculated respective RMSF values (<3 Å) which suggested the rigid structure of viral protease during simulation, except major fluctuations were recorded in the N- and C-terminal of the protein in respective complexes (Figures S4 and S5). These observations suggested that all the docked viral protease has attained the stability within 100 ns interval without significant structural distortions. Additionally, cassameridine and litseferine were docked to the viral protease’s active pocket showed fluctuations <5 Å till 80 ns and then followed by state of equilibrium. Whilst laetanine and cassythicine in respective docked complexes were logged for superior state stability and acceptable deviations <7 till end of 100 ns. Furthermore, calculated RMSF values for each ligand showed <2 Å fluctuation during the 100 ns simulation, suggested the considerable stability of docked compound on the active pocket of viral protease. However, viral protease docked with GC376 reference inhibitor showed deviation <3.5 Å, (Figure S3) and RMSF value (<3 Å) calculation supported this observation. These observations suggests that the selected phytochemical shows considerable dynamic stability with the SARS-CoV-2 M^pro^.

#### 3.3.2. Protein-Ligand Interaction Profiling

**Figure 4:**
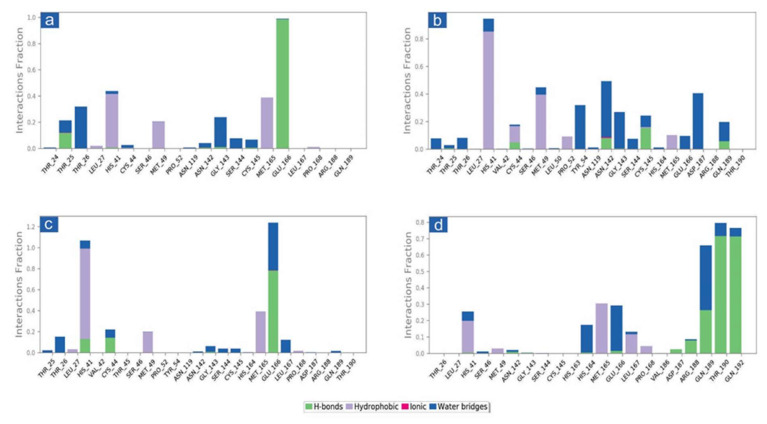
Protein-ligand interactions mapping for SARS-CoV-2 docked with potential compounds, i.e., (**a**) Cassameridine, (**b**) Laetanine, (**c**) Litseferine, and (**d**) Cassythicine, extracted from 100 ns MD simulations. Herein, values of interaction fractions >1.0 are feasible as some residues established several interactions of the similar subtype.

**Figure 5:**
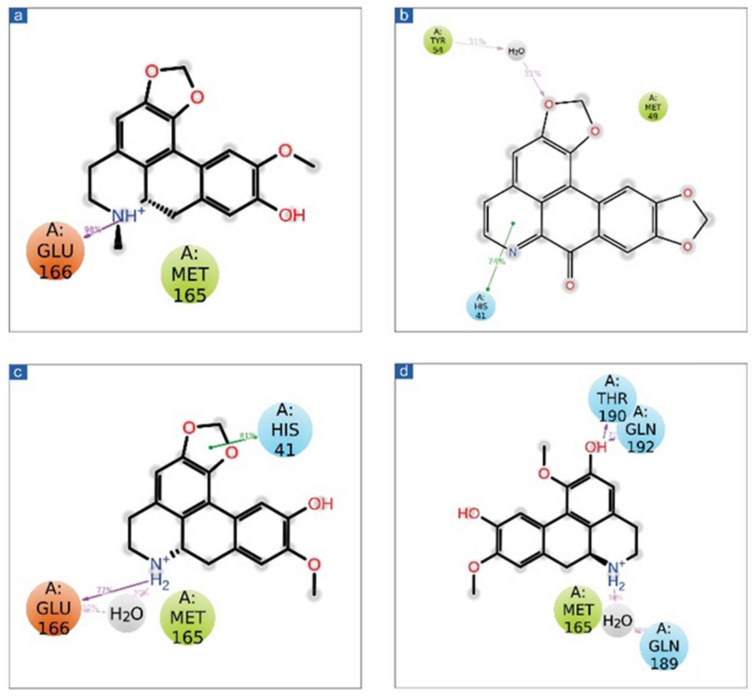
Schematic representation for docked protein-ligand complexes, viz. (**a**) Cassameridine, (**b**) Laetanine, (**c**) Litseferine, and (**d**) Cassythicine, interaction profile extracted at 30% of total 100 ns simulation interval.

The docked complexes of viral proteins with potential compounds were also considered for protein-ligand interaction profiling in hydrogen bonding, hydrophobic interactions, ionic interactions, and water bridge formation throughout a 100 percent simulation period. Remarkably, all the complexes were logged for significant encounters with the active residues of the viral protease during 100 ns simulation. For instance, in SARS-CoV-2-cassameridine, the hydrogen bond formation was exhibited by residue Glu166 for 100% of the simulation time. In contrast, His41 and Met165 residues were noted for hydrophobic interaction with the docked ligands for more than 35% of the total interaction fraction. Additionally, residues Thr26 and Gly143 demonstrated water bridge formation throughout more than 10% of the simulation interval (Figure 4a and Figure 5a). Similar to this, for 20% of the simulation interval, Cys145 in the M^pro^ -Laetanine complex of SARS-CoV-2 demonstrated hydrogen bond formation. Additionally, His41 and Met49 exhibit hydrophobic contacts at 80% and 40% of the total interaction percentage, respectively. Besides, Tyr54, Asp187 and Asn142 contribute to water bridge formation for 30% of 100 ns simulation time (Figure 4b and Figure 5b). In SARS-CoV-2 M^pro^-Litseferine docked complex, Glu166 and His41 exhibit hydrogen bond and hydrophobic interaction for 70% total interaction fraction in addition to water bridge formation (40% interaction fraction) (Figure 4c and Figure 5c). Additionally, protein-ligand contact analysis of SARS-CoV-2 M^pro^-Cassythicine complex showed a substantial contribution of Thr190 and Gln192 in hydrogen bond formation for more than 70% of total interaction fraction and Met165 contributes in hydrophobic interaction for 30% of total interaction fraction. Gln189, Glu166 and His164 residues contributes in water bridge formation (Figure 4d and Figure 5d). Interestingly, the protein-ligand mapping of the SARS-CoV-2 with the reference ligand GC376 substantially demonstrates hydrogen bond formation via Gly143, Cys145, Glu166 and Gln189 for more than 70% of total simulation period. Moreover, His41 and His164 contributes for water bridge formation for 80% of total interaction fraction (Figure S6a,b). All the phytochemical compound exhibits H-bond, hydrophobic bond and water bridge formation that contributes to stabilising the selected compounds within the target protein’s active site.

Additionally, the putative inhibitors of SARS-CoV-2 M^pro^ residues interacted inside molecules. Calculations of the SARS-CoV-2 M^pro^, cassameridine, laetanine, litseferine, and cassythicine at total intervals of 30% of 100 ns simulation demonstrated strong binding of the relevant ligands with active residues. It is intriguing to notice that all of the selected ligands displayed hydrogen bonding and pi-pi interactions, indicating that they would be stable in the viral protein’s active area. Based on examination of a 100 ns molecular dynamics simulation, docked complexes can be arranged in order of stability, namely SARS-CoV-2-cassameridine, SARS-CoV-2-litseferine, SARS-CoV-2-laetanine, and SARS-CoV-2-cassythicine.

### 3.4. Essential Dynamics and Dynamic Cross-Correlation Matrix (DCCM) Analysis

**Figure 6:**
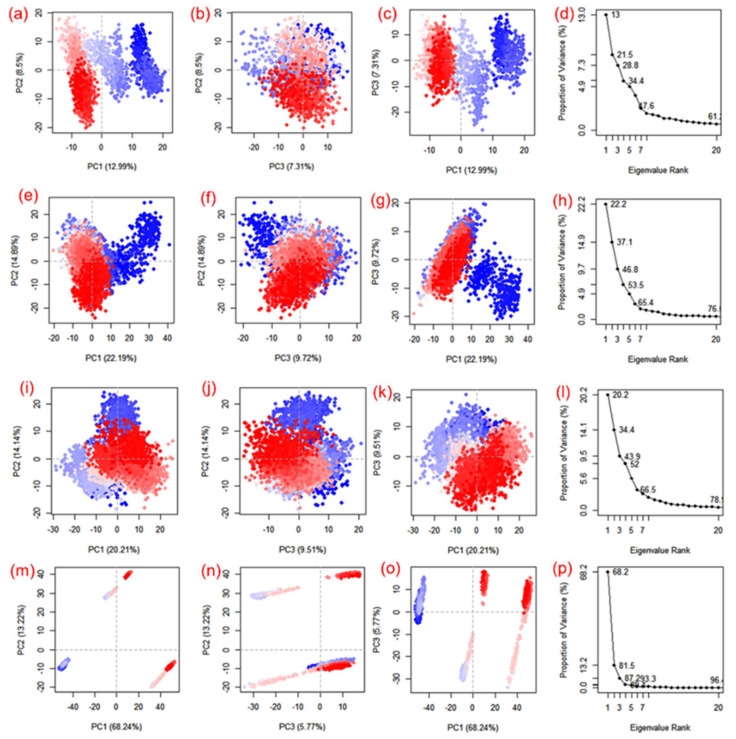
PCA analysis of SARS-CoV-2 docked simulated MD trajectories with cassameridine (**a**–**d**), laetanine (**e**–**h**), litseferine (**i**–**l**), and cassythicine (**m**–**p**). Each direction’s logged deviations in the residue location are categorised by the analogous eigenvalue’s total percentage of mean square displacements (PCs). Periodic jumps between the structural conformations taken from the 100 ns simulation trajectories are visible in the continuous colour change from blue to white to red.

Essential dynamics, also known as principal component analysis (PCA), was performed on the MD trajectories to collect the key eigenvalues to better understand the dynamics of the protein domains and residual displacements. This statistical technique is based on covariance matrices. Specifically, PCA components were taken from 100 ns for the SARS-CoV-2 M^pro^ docked with cassameridine, (b) laetanine, (c) litseferine, and (d) cassythicine. Figure 6 shows the variance (%) (eigen fraction) as a function of the 20 eigenmodes and the mean square positional fluctuations in the covariance matrix as MD trajectories. With the selected compounds of each SARS-CoV-2 M^pro^ docked system showed a sharp decline in Eigen fraction that matched the early three eigenmodes, indicating a significant degree of conformational mobility brought on by the docked ligand within the active pocket of the viral protease. After the 4th eigen value, however, a subsequent elbow point and no change in the fluctuations of the eigen fraction were found. (Figure 6). These findings suggested that SARS-CoV-2 M^pro^ exhibits significant flexibility when docked with particular compounds during the MD simulation’s early stages, which reduced flexibility. The gradual drop in the relative contribution of the eigen modes also suggested that further localised variations in SARS-CoV-2 M^pro^ docked with each molecule be added to achieve the desired stability. Therefore, it was proposed that these changes within each complex were crucial to the stability of the corresponding docked complexes.

Apart from the SARS-CoV-2 M^pro^-Cassythicine complex, the first three SARS-CoV-2 M^pro^ engine vectors that docked with each compound and were derived from the associated MD trajectory as cluster groups displayed compact and cluster motions for SARS-CoV-2 M^pro^ in the corresponding trajectory (Figure 6). Additionally, the generated plots showed that throughout the MD simulation, there were variations in the cluster distribution in each conformation. The blue to red colour gradient represents repeated jumps between the several structural positions of the docked viral protease. A corelated fluctuating motion of the viral protease during MD simulation in all of the systems under study, with the exception of SARS-CoV-2 M^pro^-Cassythicine, depicts the stiffness and stability of the associated docked complexes.

**Figure 7:**
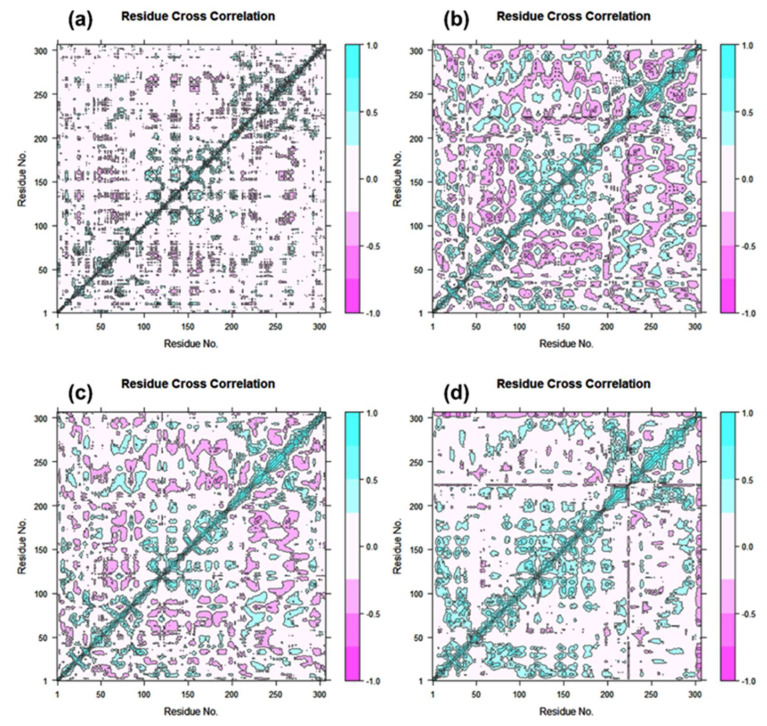
Dynamic cross correlation for Tyrosinase complexed with (**a**) Cassameridine, (**b**) Laetanine, (**c**) Litseferine, and (**d**) Cassythicine, (Note Residues labels are numbered from 1–306 as in crystal structure). During a 100 ns simulation interval, the movement of residues exhibits a positive correlation in cyan colour and a negative correlation in cyan red.

DCC matrix analysis was used to quantify the frequency of associated motions during MD simulation based on the positions of C-alpha atoms to calculate the structural dynamics changes brought about in SARS-CoV-2 M^pro^ as a result of the docked ligands’ inhibitory activity. Figure 7 displays motions with high correlation, from light blue to cyan (+1), and motions with low correlation, from light purple to red brick black (−1). Analysis of the residue cross correlation, which suggested substantial correlated motions and dynamic changes, revealed no significant correlated motions and dynamics changes in any of the systems, with the exception of complexes docked with laetanine and cassythicine. The other two complexes, however, revealed variations in the residues involved in molecular interactions with the respective ligand. The calculated results established that Laetanine and Cassythicine significantly changed the conformation of docked viral protease during the MD simulation.

Based on the structural analysis of the MD simulation results for Tyrosinase complexes with specific ligands and molecular docking, i.e., (a) Cassameridine, (b) Laetanine, (c) Litseferine, and (d) Cassythicine, we suggested that screened potential compounds holds the potential to inhibit the activity of viral protease via strong intermolecular interactions for stable docked complex formation as well disturbing the conformation of viral protease active pocket.

## 4. Conclusions

With the progression of SARS-CoV-2 infection and the lack of a prospective antiviral medicine, plant-based natural products are being investigated as a potential source for antiviral medication development. This study applied molecular docking and simulation approach to identify potential SARS-CoV-2 M^pro^ inhibitory antiviral phytomolecules derived from Lauraceae family plants. Four prominent compounds, i.e., cassameridine, laetanine, litseferine and cassythicine, were identified through virtual screening with acceptable docking scores (>−8.6 kcal/mol) belonging to plants of Laucrace family, respectively. The binding affinity, intermolecular interactions, and dynamic stability of all the respective docked complexes were further evaluated using various computational approaches against the SARS-CoV-2 M^pro^-GC376 as reference complex. A collective analysis suggested that all four selected phytomolecules posses’ significant affinity and stability within the binding pocket of SARS-CoV-2 M^pro^. Therefore, these phytomolecules can be appraised as potential anti-SARS-CoV-2 compounds and examined through in vitro experiments to assess their efficacy and potency.

## Figures and Tables

**Table 1:** The structure-based virtual screening process obtained names and characteristics of the selected phytochemical compounds against the SARS-CoV-2 M^pro^ receptor from the collected phytochemical compounds.

S.no	Pubchem ID	Compound	Molecular wt.	Origin	Docking Score (kcal/mol)
1	12302502	Cassameridine	319.3	Cassytha filiformis	−9.3
2	129371873	Laetanine	313.3	Ocotea teleiandra	−8.8
3	13891936	Litseferine	311.3	Litsea glutinosa	−8.6
4	442194	Cassythicine	325.4	Licaria sebifera	−8.6

**Table 2:** Intermolecular interaction patterns for the phytochemical compounds docked in the SARS-CoV-2 M^pro^ protein binding pocket in conformation with the active residues.

S.No.	Complex	H-Bond	Hydrophobic	Polar	π-π Stacking	Positive	Negative
1	SARS-CoV-2 M^pro^-cassameridine	Gly^143^, Glu^166^	Leu^27^, Met^49^, Tyr^54^, Cys^145^, Met^165^	Thr^25^, Thr^26^, His^41^, Asn^142^, Gln^189^	His^41^	Arg^188^	Glu^166^ Asp^187^
2	SARS-CoV-2 M^pro^-laetanine	Glu^166^	Leu^27^, Met^49^, Tyr^54^, Leu^141^, Cys^145^, Met^165^	Thr^25^, Thr^26^, His^41^, Asn^142^, Ser^144^, His^163^, His^164^ Gln^189^	--	Arg^188^	Glu^166^ Asp187
3	SARS-CoV-2 M^pro^-litseferine	His^41^ Glu^166^	Leu^27^, Met^49^, Tyr^54^, Cys^145^, Met^165^	Thr^25^, Thr^26^, His^41^, Asn^142^, Ser^144^, His^164^ Gln^189^	His^41^	Arg^188^	Glu^166^ Asp^187^
4	SARS-CoV-2 M^pro^-cassythicine	Gly^143^ Glu^166^	Leu^27^, Va^l42^, Met^49^, Tyr^54^, Cys^145^, Met^165^	Thr^25^, His^41^, Asn^142^, His^164^ Gln^189^	His^41^	Arg^188^	Glu^166^ Asp^187^
5	SARS-CoV-2 M^pro^-GC376	Phe^140^ His^163^ Glu^166^ Gln^189^	Met^49^, Tyr^54^, Phe^l40^ Leu^141^ Cys^145^, Met^165^ Leu^167^ Pro^168^ Ala^191^	His^41^, Asn^142^, Ser^144^, His^163^, His^164^ His^172^ Gln^189^ Thr^190^, Gln_192_	--	Arg^188^	Glu^166^ Asp^187^

## Data Availability

The datasets used and analysed during the current study are available from the corresponding author at reasonable request.

## Supplementary Materials

The following supporting information can be downloaded at: https://www.mdpi.com/article/10.3390/v14122783/s1, Table S1. List of screened compounds using MTiopen screen against the SARS-CoV-2 M^pro^. Figure S1. 2d image of the reference ligand GC376. Figure S2. Redocking and Intermolecular interaction analysis. Figure S3. RMSD plots for SARS-CoV-2 Mpro docked with reference compound GC376 extracted from 100 ns MD simulation. Figure S4. RMSF plots for SARS-CoV-2 M^pro^ (a) Cassameridine, (b) Laetanine, (c) Litseferine, (d) Cassythicine, and (e) GC376- reference compound for the 100 ns MD simulation time. Figure S5. RMSF plots for docked complex and reference (a) Cassameridine, (b) Laetanine, (c) Litseferine, (d) Cassythicine, and (e) GC376 for the 100 ns MD simulation time. Figure S6. (a) Protein-ligand interactions mapping for SARS-CoV-2 docked with reference compound GC376 inhibitor. (b) Schematic representation for SARS-CoV-2- GC376 complex for the 100 ns MD simulation time.
