# Supplementary material for: Exploration of Potent Antiviral Phytomedicines from Lauraceae Family Plants against SARS-CoV-2 Main Protease
Source: Viruses. 2022 Dec 14;14(12):2783. doi: 10.3390/v14122783 (PMC9785681; doi:10.3390/v14122783)
Supplement: Supplementary file 1 [file viruses-14-02783-s001.zip › viruses-2036119-supplementary.pdf]

Exploration of Potent Antiviral Phytomedicines from Lauraceae Family Plants against SARS-CoV-2 Main Protease

S1. Results and Discussion

S1.1. Structure based virtual screening

Table S1. List of screened compounds using MTiopen screen against the SARS-CoV-2 M^{pro}.

S.no.	Compound	Model ID	Energy	nRot
1	12302502	1	-9.3	0
2	129371873	1	-8.8	0
3	13891936	1	-8.6	2
4	442194	1	-8.6	2
5	160502	1	-8.5	2
6	15690954	1	-8.2	2
7	101300	1	-8.2	2
8	489948	1	-8.1	4
9	177744	1	-8.1	2
10	10144	1	-8	0
11	44578390	1	-7.9	0
12	12305138	1	-7.9	2
13	168363	1	-7.9	2
14	14262868	1	-7.8	4
15	14539910	1	-7.8	4
16	14539911	1	-7.8	4
17	197018	1	-7.7	1
18	21222	1	-7.7	4
19	24982202	1	-7.7	16
20	181681	1	-7.7	7
21	16754	1	-7.6	0
22	160500	1	-7.6	4
23	73337	1	-7.6	4
24	439653	1	-7.6	0
25	10154	1	-7.6	4
26	133323	1	-7.6	4
27	10042942	1	-7.6	0
28	160487	1	-7.5	5
29	22179	1	-7.5	4
30	31415	1	-7.4	4
31	179491	1	-7.4	4
32	100067	1	-7.4	8
33	12313549	1	-7.4	4
34	65631	1	-7.4	2
35	101289740	1	-7.3	4
36	440595	1	-7.3	5

37	196885	1	-7.3	4
38	101140028	1	-7.3	4
39	53266	1	-7.3	5
40	16573	1	-7.2	4
41	91884708	1	-7.2	4
42	282014	1	-7.1	5
43	157129	1	-7.1	5
44	10153	1	-7.1	4
45	10573801	1	-7.1	4
46	10405046	1	-7.1	3
47	5280794	1	-7.1	6
48	5742590	1	-7.1	13
49	131751584	1	-7	5
50	12313923	1	-7	3
51	73117	1	-6.9	0
52	5484202	1	-6.9	6
53	57525693	1	-6.9	7
54	10717392	1	-6.8	4
55	14526072	1	-6.8	5
56	222284	1	-6.8	7
57	165225	1	-6.7	9
58	12016618	1	-6.7	4
59	15690955	1	-6.6	5
60	10143	1	-6.6	4
61	442876	1	-6.6	8
62	6432404	1	-6.6	0
63	145858	1	-6.5	1
64	91884834	1	-6.4	9
65	90785	1	-6.3	2
66	12306048	1	-6.1	1
67	441005	1	-6	1
68	10398656	1	-6	2
69	91457	1	-5.8	2
70	10657	1	-5.8	1
71	92762	1	-5.6	2
72	6432005	1	-5.5	2
73	10910653	1	-5.4	0
74	76312534	1	-5.4	5
75	44566761	1	-5.4	3
76	92138	1	-5.4	4
77	76312534	1	-5.4	5
78	12306052	1	-5.4	1
79	1549026	1	-5.3	6
80	91354	1	-5.3	0
81	22559443	1	-5.3	0
82	9548705	1	-5.3	1

83	6432312	1	-5.2	2
84	1549025	1	-5.1	6
85	5281515	1	-5.1	0
86	5280435	1	-5.1	14
87	643779	1	-5	4
88	7439	1	-5	0
89	6918391	1	-4.9	3
90	3314	1	-4.9	4
91	5282743	1	-4.9	14
92	11463	1	-4.9	0
93	5316956	1	-4.8	10
94	11142	1	-4.8	0
95	445639	1	-4.8	16
96	5281520	1	-4.8	0
97	7439	1	-4.8	0
98	643820	1	-4.8	5
99	17100	1	-4.8	2
100	6549	1	-4.7	5
101	5312399	1	-4.7	12
102	637566	1	-4.7	5
103	22311	1	-4.7	0
104	11230	1	-4.6	2
105	7463	1	-4.6	1
106	5280450	1	-4.6	15
107	14896	1	-4.6	0
108	5281553	1	-4.5	3
109	101629835	1	-4.5	1
110	8842	1	-4.5	6
111	11005	1	-4.5	13
112	170833	1	-4.5	2
113	638011	1	-4.4	4
114	7794	1	-4.4	5
115	31253	1	-4.4	4
116	2969	1	-4.4	9
117	18818	1	-4.4	1
118	6654	1	-4.3	0
119	5320250	1	-4.3	3
120	2758	1	-4.2	0
121	985	1	-4.2	15
122	3893	1	-4.2	11
123	6616	1	-3.9	0

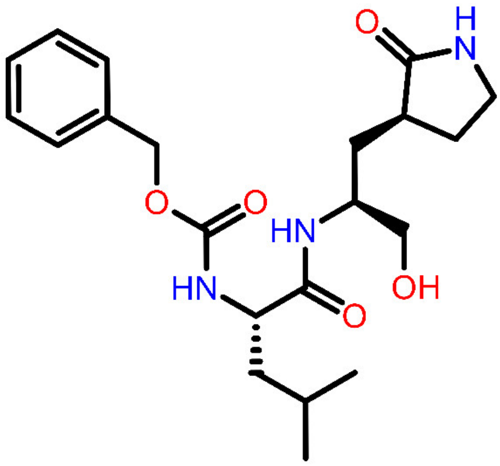

Figure S1. 2d image of the reference ligand GC376.

Redocking and Intermolecular interaction analysis

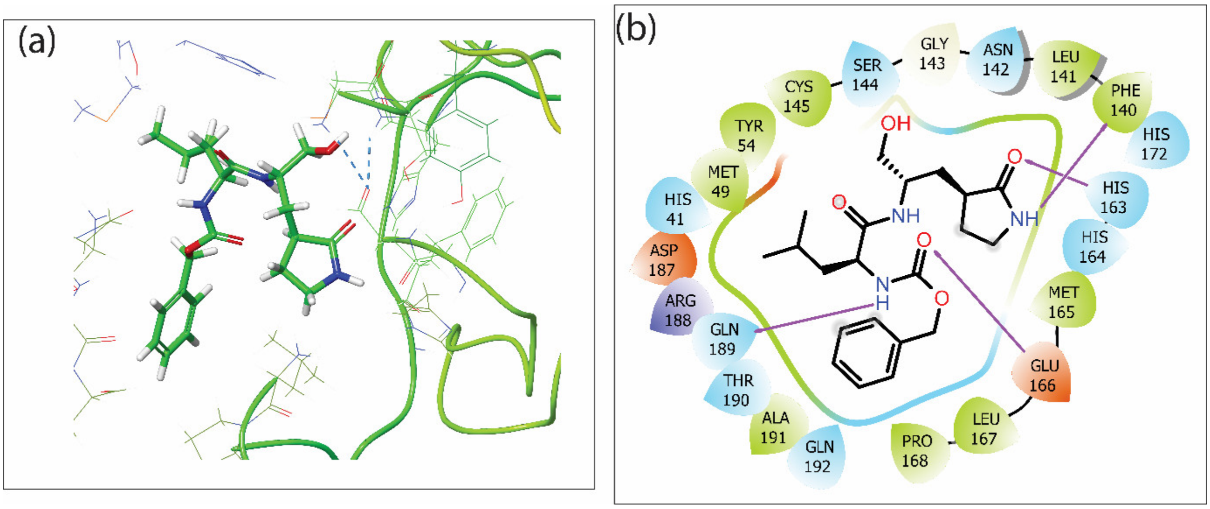

Figure S2. (**a**) 3D and 2D representation of SARS-CoV-2 docked complexes exhibiting intermolecular interactions with reference compound GC376. (**b**) In 2D interaction profiles, hydrogen bonds (pink arrows), hydrophobic (green), polar (blue), negative (red), positive (violet) and glycine (grey) interactions are shown to be involved between the docked protein-ligand complexes.

Molecular dynamics simulation analysis.

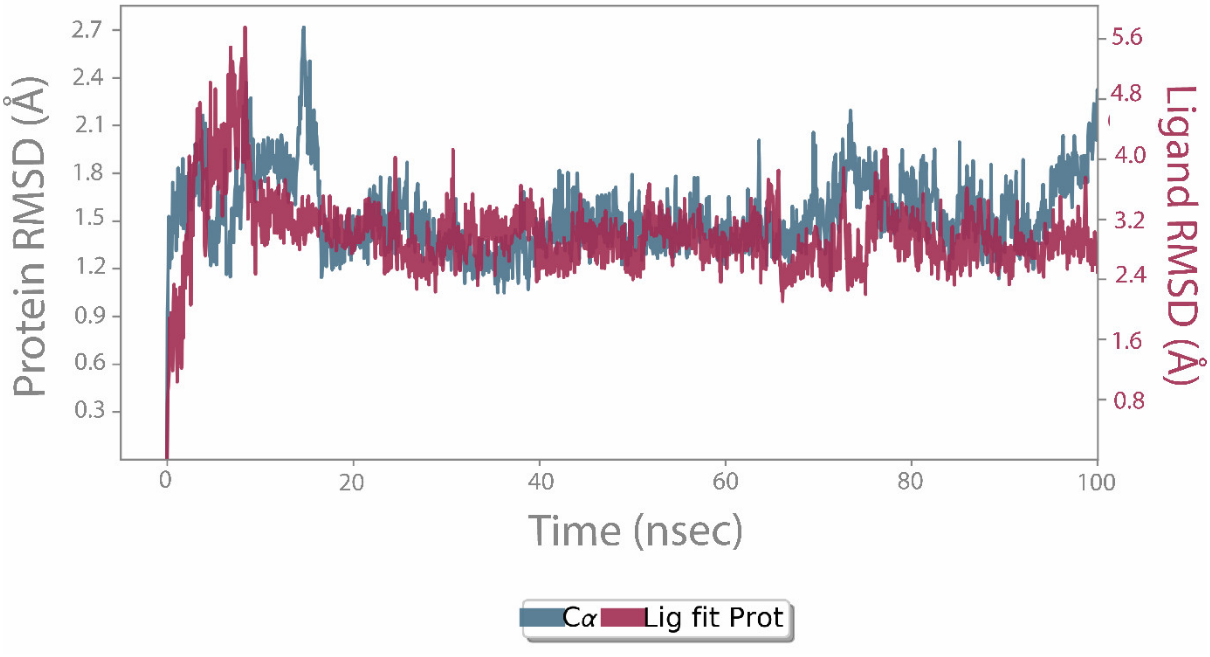

Figure S3. RMSD plots for SARS-CoV-2 M^{pro} docked with reference compound GC376 extracted from 100 ns MD simulation.

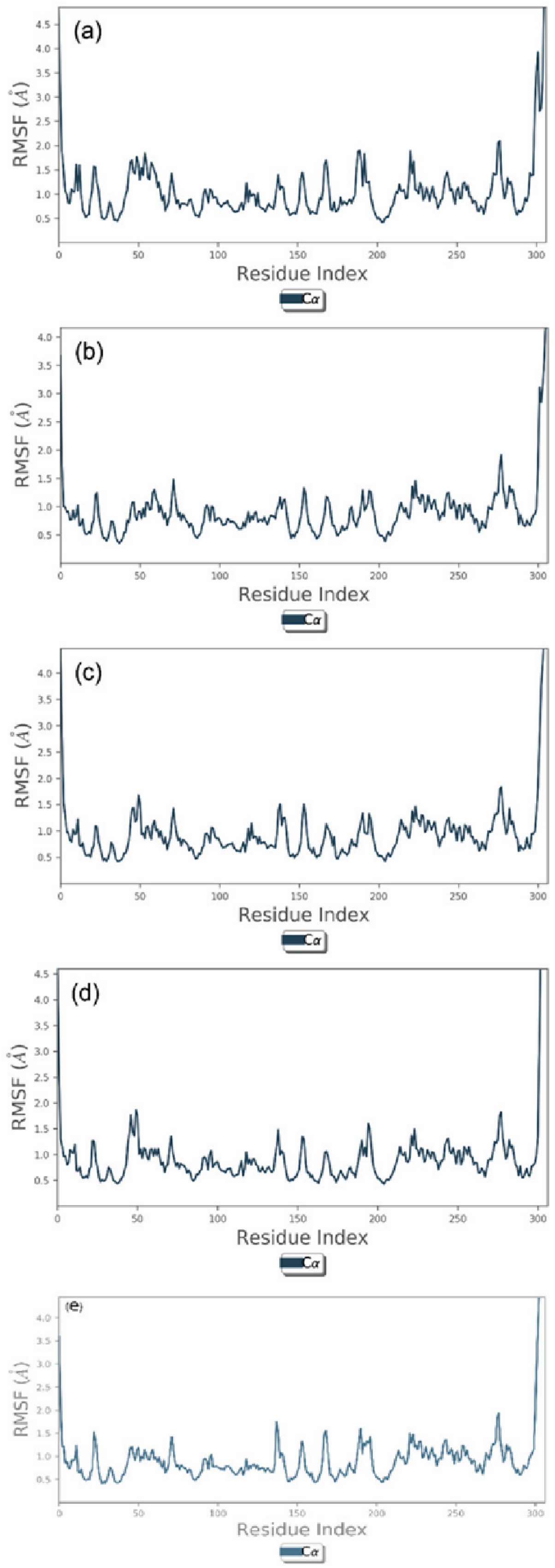

Figure S4. RMSF plots for SARS-CoV-2 M^{pro} (**a**) Cassameridine, (**b**) Laetanine, (**c**) Litseferine, (**d**) Cassythicine, and (**e**) GC376- reference compound for the 100 ns MD simulation time.

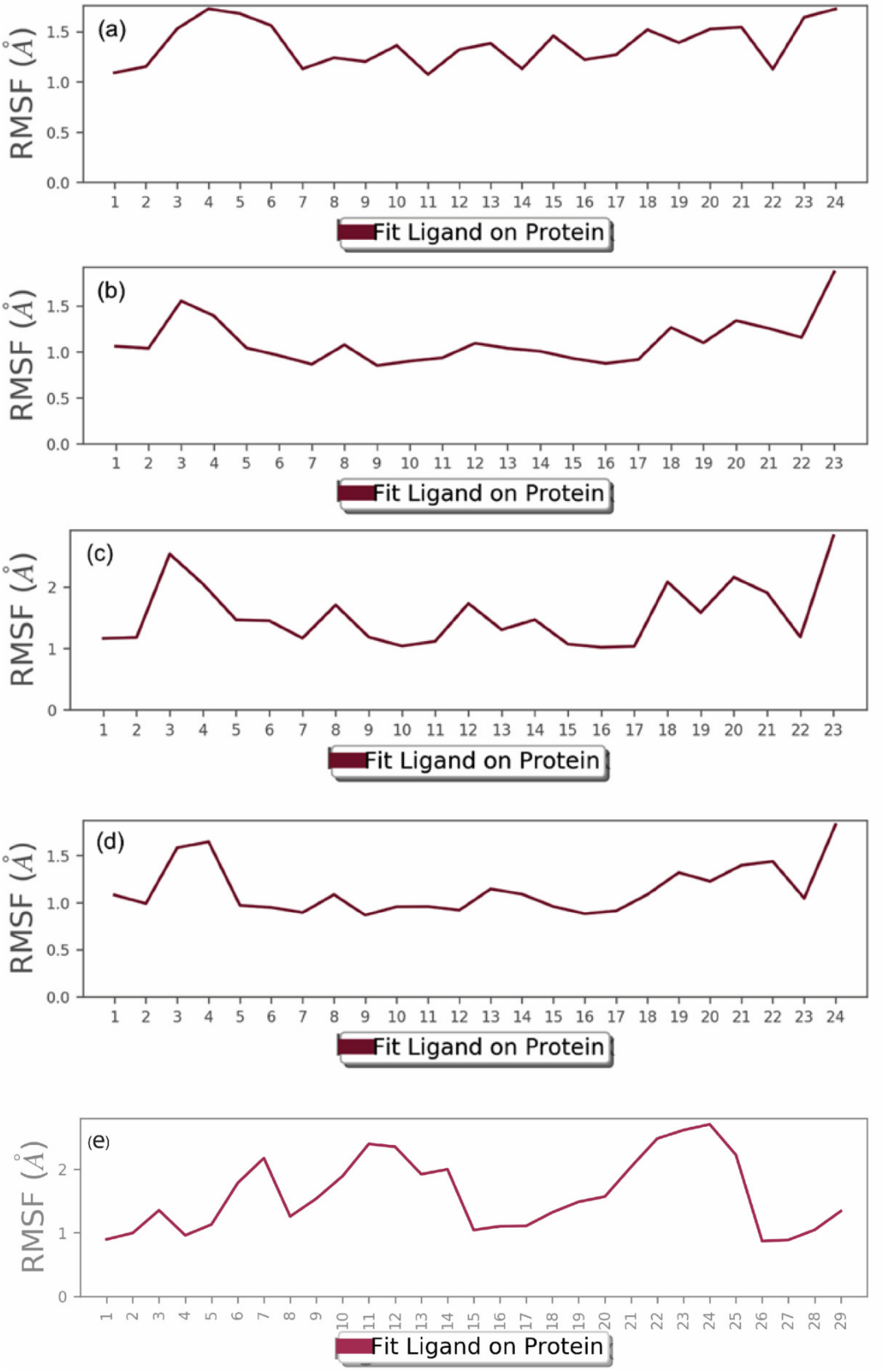

Figure S5. RMSF plots for docked complex and reference (**a**) Cassameridine, (**b**) Laetanine, (**c**) Litseferine, (**d**) Cassythicine, and (**e**) GC376 for the 100 ns MD simulation time.

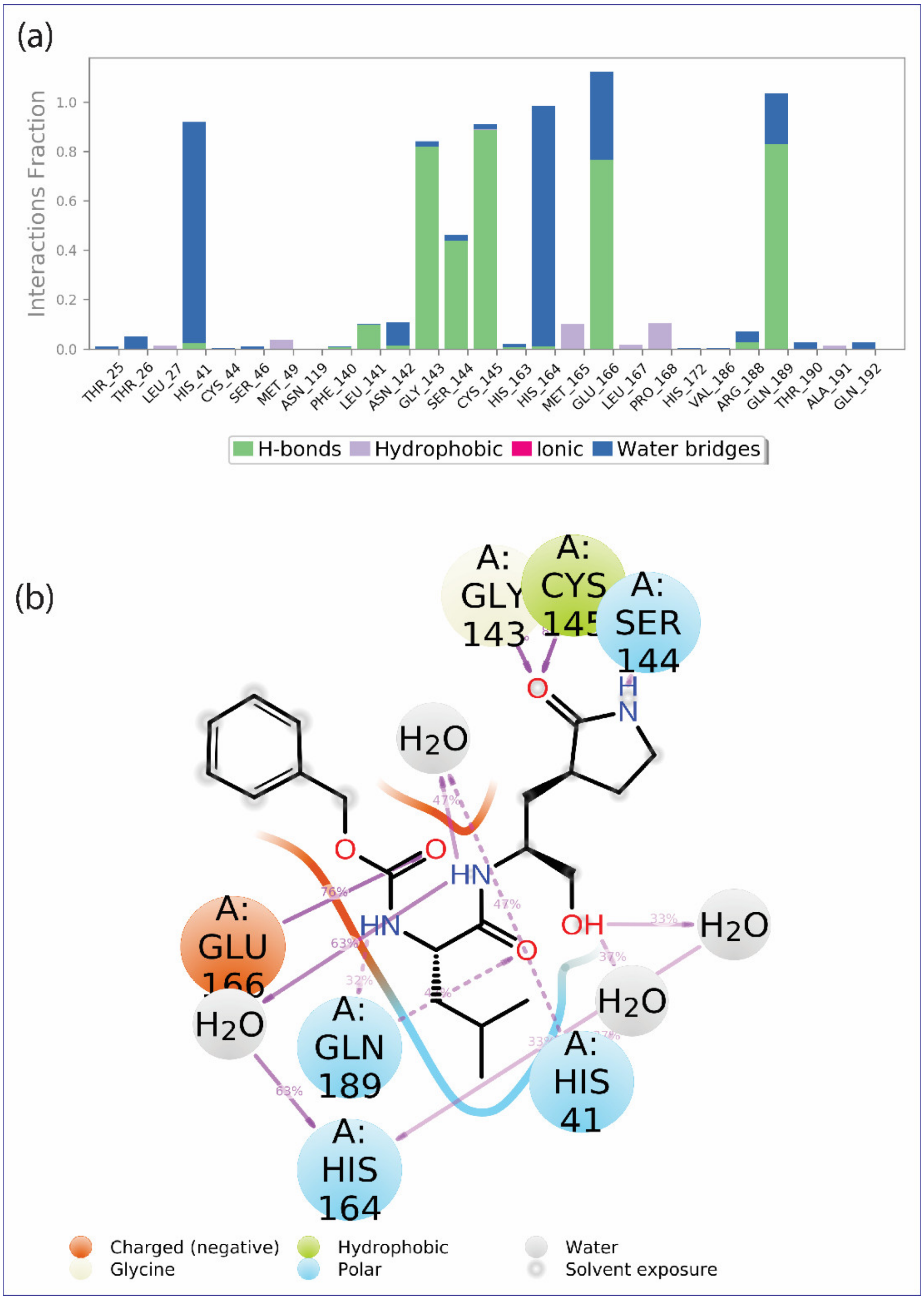

Figure S6. (**a**) Protein-ligand interactions mapping for SARS-CoV-2 docked with reference compound GC376 inhibitor. (**b**) Schematic representation for SARS-CoV-2- GC376 complex for the 100 ns MD simulation time.
